# The Altered Functional Connectivity With Pain Features Integration and Interaction in Migraine Without Aura

**DOI:** 10.3389/fnins.2021.646538

**Published:** 2021-03-04

**Authors:** Zilei Tian, Tao Yin, Qingqing Xiao, Xiaohui Dong, Yunhong Yang, Menglin Wang, Guodong Ha, Jiyao Chen, Fanrong Liang, Fang Zeng, Lei Lan

**Affiliations:** ^1^Acupuncture and Tuina School/The 3rd Teaching Hospital, Chengdu University of Traditional Chinese Medicine, Chengdu, China; ^2^Acupuncture and Brain Science Research Center, Chengdu University of Traditional Chinese Medicine, Chengdu, China; ^3^Key Laboratory of Sichuan Province for Acupuncture and Chronobiology, Chengdu, China

**Keywords:** migraine without aura, fMRI, resting-state, functional connectivity, pain discrimination, pain integration, moderating effects

## Abstract

**Introduction:**

Migraine without aura (MwoA) is a primary type of migraine, a common disabling disorder, and a disabling neurological condition. The headache is a complex experience, a common form of pain, in which multiple sensory information dimensions are combined to provide a unified conscious event. Migraine ictal have unique neuroimage biomarkers, but the brain is also affected during the inter-ictal phase. According to the current studies, a hypothesis was constructed that the altered integration of pain spatial and intensity information impacts headache intensity in the inter-ictal period.

**Methods:**

In this study, we applied theory-based region-to-region functional connectivity (FC) analyses to compare the differences in resting-state FC between MwoA participants and healthy controls with the pain integration hypothesis. After the correlation matrices between FC edges and clinical symptoms were constructed, the moderating effect and simple slope tests were investigated to explain whether and how the dysfunction of pain features discrimination affects the clinical symptoms.

**Results:**

Functional connectivity analyses showed significantly decreased FC edges between the left dorsolateral superior frontal gyrus (SFGdor) and left insula, and an increased FC edge between the left SFGdor and bilateral angular gyrus. The correlation matrix showed no significant correlation between significantly altered FC edge and headache duration, frequency, Zung self-rating anxiety scale, and Zung self-rating depression scale. Only one significantly altered edge in the MwoA condition was significantly correlated with headache intensity. Moderating Module 1 and 2 manifested the moderator variable (altered rs-FC edge) moderated the link between the normal edges and headache intensity.

**Conclusion:**

The pain features integration processes in migraineurs vary from HCs, related to the clinical symptoms during a migraine attack. Moreover, the clinical symptoms will be affected by one or more discrimination modules. And the spatial or intensity discrimination modules have a higher impact when combined with another module on clinical symptoms than the single module.

## Introduction

Migraine is one of the main leading causes of disability worldwide (Stovner et al., 2018), and the most prevalent disorder (Disease et al., 2017) caused substantial burdens and impacts (Leonardi and Raggi, 2019). Migraine without aura (MwoA) is a primary type of migraine (Noseda and Burstein, 2013). The typical features of MwoA include a lasting four to 72 h recurrent headache disorder with unilateral location, pulsating quality, and moderate or severe intensity (Headache Classification Subcommittee of the International Headache Society, 2018).

Diverse neuroimaging techniques were taken to investigate the neuropathic patterns of neurology over the past few decades (Lovati et al., 2016). Functional magnetic resonance imaging (fMRI), especially the resting-state fMRI (rs-fMRI), has been widely used for understanding migraine-related pathophysiology (Tedeschi et al., 2013; Chong et al., 2016; Russo et al., 2017), mechanisms (Schwedt et al., 2015), and biomarkers (Skorobogatykh et al., 2019). These studies demonstrated that pain processing, emotional processing, cognitive processing, and pain modulation are entirely involved in migraine mechanisms, which further provided a solid framework on migraine: (1) migraine neuropathology contains brain dysfunction: especially the atypical pain processing; (2) the altered functional connectivity (FC) or networks developing in sequence.

**FIGURE 1:**
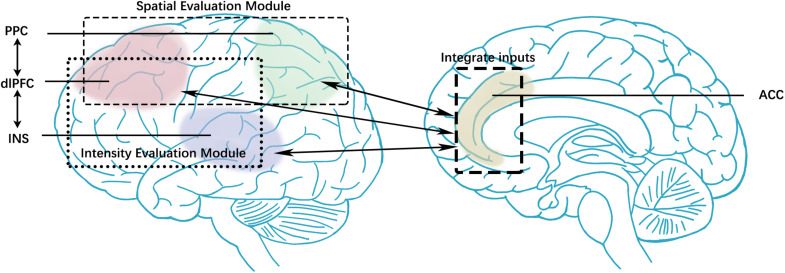
The pain integration hypothesis included two components: intensity and spatial evaluating modules. Intensity information is processed by a ventrally processing module involving the insula, bilateral portions of the prefrontal cortex, and cingulate cortex. Spatial information is processed by a dorsally processing module that involves the posterior parietal cortex, dorsolateral prefrontal cortex, and anterior cingulate cortex. The anterior cingulate cortex takes participated in both intensity and spatial discrimination modules. PPC, posterior parietal cortex; dlPFC, dorsolateral prefrontal cortex; INS, insula; ACC, anterior cingulate cortex.

Present studies spotted that the attribute and intensity of stimuli in one sensory system could moderate symptoms in other sensory domains (Schwedt et al., 2015). The abnormal activation of the painful, olfactory, and visual stimulation present in migraineurs is widely recognized. These features set migraine apart from other neurological disorders and help improve diagnosis accuracy (Tu et al., 2020) and predict treatment efficacy (Yin et al., 2020). Therefore, “a pain processing network” offered a holistic perspective compared with “a separate migraine generator” on migraine research. Our previous studies (Li et al., 2016) investigated the altered FC between periaqueductal gray and medial prefrontal cortex (mPFC) and the association with headache intensity, which indicated that the pain circuit is a part of MwoA mechanisms. The insights from other investigations on the default-mode network (DMN), central executive network (CEN), and other networks had already provided perspectives on attention, working memory, and visual processing on migraine mechanism (Lakhan et al., 2013; Colombo et al., 2015; Demarquay and Mauguiere, 2016). However, the discrimination of sensory features and integrating pain information in migraine has been little studied. We hypothesized that MwoA patients have inter-ictal abnormalities in pain integration functions that affect clinical headache symptoms. The pain integration hypothesis included two components: intensity and spatial evaluating modules (Figure 1). Intensity information is processed by a ventrally processing module involving the insula (INS), bilateral portions of the prefrontal cortex, and cingulate cortex. Spatial information is processed by a dorsally processing module that involves the posterior parietal cortex (PPC), dorsolateral prefrontal cortex (dlPFC), and anterior cingulate cortex (ACC). The ACC takes participated in both intensity and spatial discrimination modules (Oshiro et al., 2009). Since pain is a complex experience, multiple sensory information needs to be combined to finish the sensation of pain (Huang et al., 2020), and changes in one of these components will affect the pain sensation entirely. This study pays attention to the integration of pain features and the interactions between modules.

This study aimed to understand whether and how the pain features integration and interaction in migraine patients affect headache symptoms with FC analysis and moderating effect analysis. Firstly, the differences in resting-state functional connectivity (rs-FC) between MwoA participants and healthy controls (HCs) were acquired with the region-of-interest (ROI)-to-ROI method. After the correlation matrix between rs-FC and clinical measures was calculated, moderating effects and simple slope tests were adapted to verify the moderating modules based on the pain integration hypothesis.

## Materials and Methods

### Participants

Sixty MwoA participants and 60 HCs were enrolled from the outpatient department of 3rd Teaching Hospital, the campus of Chengdu University of Traditional Chinese Medicine, and local advertisements. All participants signed written consent. The recruitment started in June 2011 and ended in November 2013.

Inclusion criteria of MwoA participants were as follows: (1) 17–45 years old and right-handed; (2) matched the diagnosis criteria of MwoA based on the International Classification of Headache Disorders for MwoA (2nd edition) (Headache Classification Subcommittee of the International Headache Society, 2004); (3) had a migraine duration of at least 6 months; (4) had at least one headache attack per month in the past 3 months; (5) had not taken any prophylactic medicine for headache in the past 3 months. The exclusion criteria of MwoA participants included: (1) alcohol or drug abusers; (2) pregnant or lactating women; (3) suffered from psychiatric, neurologic, cardiovascular, respiratory, or renal illnesses; (4) had any other type of headache or a history of head trauma with loss of consciousness; (5) MRI contraindications such as claustrophobia.

Healthy controls were enrolled in the campus of Chengdu University of Traditional Chinese Medicine. The inclusion criteria were as follows: (1) 17–45 years old and right-handed; (2) free from any chronic pain conditions or original diseases. The exclusion criteria included: (1) had a history of head trauma with loss of consciousness; (2) pregnancy or lactation women.

Each participant underwent a detailed medical history taking, physical examination, and laboratory examination to exclude potential organic disease carriers.

The case-control matching analysis was used to match demographic characteristics between the MwoA participants and the HCs, implemented in SPSS Version 25.0 (IBM Corp., Armonk, NY, United States). The MwoA patients with not matched HCs would not be analyzed. Statistically significant Pearson correlations were considered with *p*-uncorrected values lower than 0.05, two sides.

### Clinical Symptom Evaluation

The clinical symptom was evaluated with headache intensity and frequency recorded on headache diaries (Tfelt-Hansen et al., 2000). Patients were asked to record headache diaries during a 4-week observation period. The headache intensity evaluation was based on a 0–10 visual analog scale (VAS) score, in which “0” points indicating no pain and “10” points indicating the worst pain imaginable. The average VAS score for each migraine attack during the observation period was defined as headache intensity. The headache frequency was evaluated with the number of migraines separated by pain-free intervals of at least 48 h during the 4-week observation period. The Zung self-rating anxiety scale (SAS) and Zung self-rating depression scale (SDS) was used to evaluate the emotional status of MwoA participants.

### MRI Data Collection

MRI scans were performed at the end of the observation period. All MwoA patients were migraine-free for at least 72 h at the time of the MRI scans. MRI data were acquired with a 3.0T magnetic resonance scanner (Siemens 3T Trio Tim, Munich, Germany) with an 8-channel phase-array head coil at the West China Hospital MRI center. Participants were asked to stay awake and keep their heads still during the scan, with their eyes closed and ears plugged. The T1 images were obtained with a fast spoiled gradient recalled sequence (Parameters: slice thickness = 1 mm; repetition time = 1900 ms; echo time = 2.26 ms; field of view read = 256 mm). The blood-oxygen-level-dependent (BOLD) images were obtained with echo-planar imaging parameters: slice = 30; total volumes: 180; slice thickness = 5 mm; repetition time = 2000 ms; echo time = 30 ms; field of view read = 240 mm).

### MRI Data Processing

The CONN functional connectivity toolbox (19.c) (Whitfield-Gabrieli and Nieto-Castanon, 2012) implemented in MATLAB 9.4 (The Mathworks, Inc., Natick, MA, United States) and statistical parametric mapping analysis package^1^ version SPM12 (7487) was used to preprocess MRI images, conduct ROI-to-ROI FC analysis, and visualize results. fMRI data would be preprocessed with the following steps: (1) convert to NIFTI 4D image; (2) functional images realignment and unwarp; (2) translate functional images coordinates; (3) slice-timing correction; (4) outlier detection with the artifact detection tools based identification of outlier scans for scrubbing; (5) segmentation (gray matter/white matter/cerebrospinal fluid) and normalization; (6) translate structural coordinates; (7) structural images segmentation and normalization; (8) functional images smoothing (half-peak full-width Gaussian kernel = 4 mm). The 97th percentile standardized sampling was used for the head motion outlier correction based on the artifact detection tools using the default setting. The default tissue probability map was used for segmentation, normalization, and resampling: structural phase resolution = 1 mm; functional phase resolution = 2 mm.

### ROI Selection

According to the pain integration hypothesis (Figure 1), the spatial features of stimuli are processed by a dorsal stream involving the PPC and dlPFC. The pain intensity (non-spatial features) discrimination related to bilateral INS and PFC. The ACC is involved in both spatial and intensity evaluating modules as a sensory monitor. Thus, we selected the bilateral dlPFC, bilateral mPFC, bilateral PPC, bilateral ACC, and bilateral INS as our ROIs based on the pain integration hypothesis. These ROIs were defined by automated anatomical labeling atlas (Rolls et al., 2015), included bilateral dorsolateral superior frontal gyrus (SFGdor), bilateral middle frontal gyrus (MFG), bilateral insula, bilateral anterior cingulate and paracingulate gyri, bilateral superior parietal gyrus (SPG), bilateral inferior parietal gyrus (IPL), bilateral supramarginal gyrus (SMG), bilateral angular gyrus (ANG) (Supplementary 1).

### Functional Connectivity Analysis

The BOLD signal units were analyzed using the percent signal change method. After the ROI-level time series was imported, linear regression was used to denoise confounding effects, including aCompCor (Behzadi et al., 2007), realignment effect, scrubbing parameters, and main condition effect. The band-pass filter (0.01–0.08 Hz) and linear detrending were applied after regression. Quality assurance was checked with the plots report after denoising. The hemodynamic response function weighted generalized linear module was used to obtain the bivariate correlation between ROIs.

The between-group comparison of FC was conducted with a two-sample *t*-test. Thresholds were defined using an alternative setting for ROI-based inferences (parametric multivariate statistics): *p*-values lower than 0.05 at cluster-level (false discovery rate correlated *p*-value with multi-voxel pattern analysis omnibus test); connection threshold at uncorrected *p*-values lower than 0.01. The default criterion aimed to select those significant edges (with larger effects than we could reasonably expect under the null hypothesis) among all ROIs. Together with a *post hoc* threshold to help characterize the pattern of individual connections that show some of the largest effects from each significant ROI.

### Moderating Effect and Simple Slope Tests

Moderating effect and simple slope tests would be performed in the following steps: (1) establish moderating effect hypotheses; (2) calculate correlation matrices; (3) construct moderating conceptual and statistical modules; (4) conduct moderating effect and simple slope tests.

According to the pain integration hypothesis, the pain features integration processes in migraineurs vary from HCs, related to the clinical symptoms during a migraine attack. FC manifests pain evaluation functions at the neural network level, every edge of rs-FC and clinical symptoms will be used as variables to testing the hypothesis. The rs-FC edges with ACC would be defined as the variable of moderating effect tests due to the central role of ACC on pain integration (Figure 1). Based on the general pain integration hypothesis, the pain integration functions would be investigated by different modules. Moderating effect hypothesis 1 indicates that clinical symptoms will be affected by one discrimination function. Hypothesis 2 suggests that clinical symptoms will be affected by both spatial and intensity discrimination modules.

Correlation matrices were constructed using MATLAB. The correlation matrices, adapted between every single edge and all 120 edges and clinical measures, would be constructed with partial correlation analysis to exclude covariate confounding effects. The age and gender were set as controlling variables.

The moderating effect and simple slope tests were conducted using Hayes PROCESS macro (Hayes, 2017) implemented in SPSS. All variables were centered and standardized (Kromrey and Foster-Johnson, 1998) before moderating effect tests being performed. The clinical symptoms were set as *Y* variable, and FC values were set as *X* or *W* variable due to the FC analysis and pain integration hypothesis. The interaction between continuous predictors *X* and *W* will be tested using simple slope tests adapted when moderating effect *p*-values are lower than 0.05 (Aiken and West, 1991). Conditioning values using the “−1 SD, mean, +1 SD” option with the “Johnson-Neyman output” option (Johnson and Neyman, 1936; Hayes, 2017). Statistically significant was considered with *p*-values lower than 0.05, two sides.

## Results

### Demographic and Clinical Data

Fifty-two MwoA patients and 60 HCs finished the MRI scan. After case-control matching analysis, four MwoA participants were excluded due to could not be matched with age or gender. Forty-eight MwoA participants and 48 age and sex-matched HCs were finally analyzed. We found no statistical difference among the HCs and MwoA patients in gender, age, height, and weight. MwoA patients had an average headache duration of 64.38 months, an average headache intensity of 5.575, and an average headache frequency of 5.917 times per month (Table 1).

**TABLE 1:** Baseline characteristics between HC and MwoA group.

	Healthy controls group (*n* = 48)	MwoA participants group (*n* = 48)	*P*-value
Female *n* (%)	35 (72.917)	35 (72.917)	1
Age (years) Mean ± SD	21.17 ± 0.859	21.23 ± 1.789	0.828
Height (cm) (Mean ± SD)	162.60 ± 7.043	161.48 ± 8.805	0.491
Weight (kg) (Mean ± SD)	52.396 ± 7.626	54.302 ± 8.970	0.265
Duration (month) (Mean ± SD)	–	64.38 ± 34.376	–
Headache intensity (Mean ± SD)	–	5.575 ± 1.082	–
Headache frequency (Mean ± SD)	–	5.917 ± 3.746	–
SAS (Mean ± SD)	–	45.406 ± 7.859	–
SDS (Mean ± SD)	–	45.276 ± 11.012	–

*MwoA, migraine without aura; SAS, Zung self-rating anxiety scale; SDS, Zung self-rating depression scale. Chi-square tests were applied for gender comparison, and two-sample *t*-tests were applied for the rest comparisons between MwoA participants and HCs.*

### MRI Data Processing and ROI-to-ROI Functional Connectivity Analyses

**FIGURE 2:**
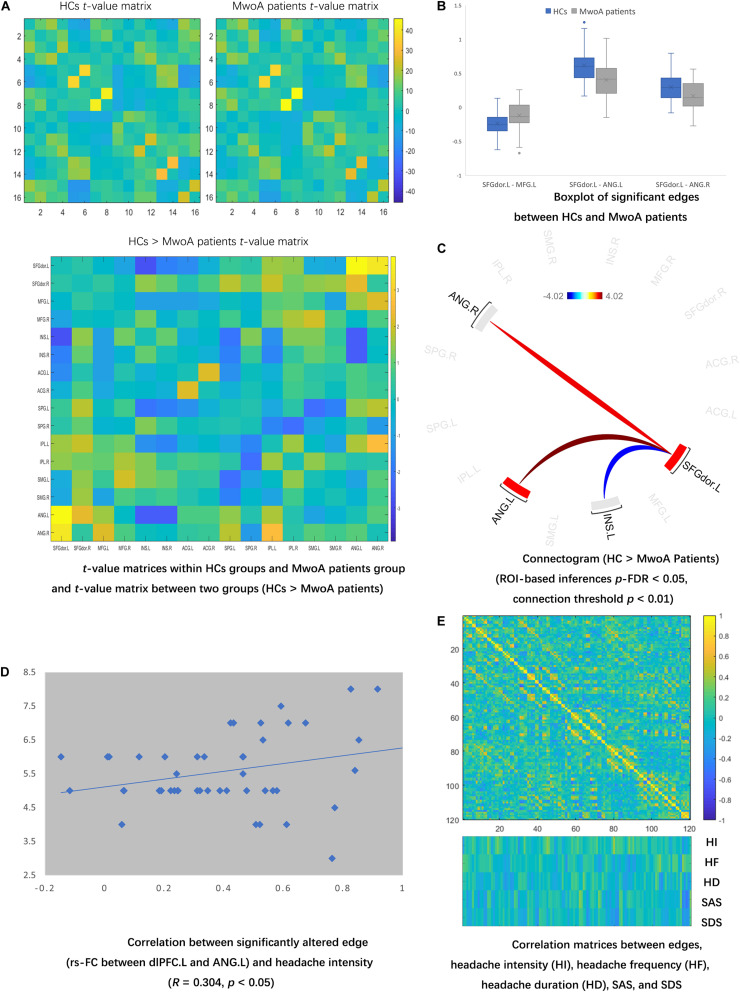
Altered rs-FC in MwoA patients and the correlation matrices of edges and clinical symptoms. **(A)** Compared with HCs, MwoA patients manifested lower rs-FC in edges between INS and SFGdor, SPG, ANG; and edges between SPG and IPL, SMG. MwoA patients showed higher rs-FC edges between ANG and SFG.dor, MFG, IPL; and edges between bilateral ACC. (uncorrected *p*-value lower than 0.05). **(B)** There were significantly lower rs-FC edges between the SFGdor.L and INS.L compared with HCs. And significantly higher rs-FC edge between the dlPFC.L and bilateral ANG than HCs. The boxplot showed the distribution of rs-FC values within and between-group. **(C)** The edges between ANG and SFGdor were significantly higher than HCs, and an edge between SFGdor and INS was significantly lower in MwoA conditions. (FDR-corrected p-value lower than 0.05) **(D)** The correlation matrix showed no significant correlation between significantly altered FC edge and headache duration, frequency, SAS, and SDS. **(E)** Six edges significantly correlated with headache intensity, four edges significantly correlated with headache frequency, five edges significantly correlated with headache duration, 12 edges significantly correlated with SAS, and six edges significantly correlated with SDS. rs-FC, resting-state functional connectivity; L, left hemisphere; R, right hemisphere; SFGdor, dorsolateral superior frontal gyrus; MFG, middle frontal gyrus; INS, insula; ACG, anterior cingulate gyri; SPG, superior parietal gyrus; IPL, inferior parietal gyrus; SMG, supramarginal gyrus; ANG, angular gyrus; HI, headache intensity; HD, headache duration; HF, headache frequency; SAS, Zung self-rating anxiety scale; SDS, Zung self-rating depression scale.

Quality assurance was checked before FC analyses, and no MwoA patients or HCs were excluded depend on the number of volumes in which head position was 0.5 mm different from adjacent volumes was more than 20% (Power et al., 2012; Supplementary 2). Compared with HCs, MwoA patients manifested lower rs-FC in edges between INS and SFGdor, SPG, ANG; and edges between SPG and IPL, SMG. MwoA patients showed higher rs-FC edges between ANG and SFG.dor, MFG, IPL; and edges between bilateral ACC (uncorrected *p*-value lower than 0.05) (Figure 2A). There were significantly lower rs-FC edges between the SFGdor.L and INS.L compared with HCs. And significantly higher rs-FC edge between the dlPFC.L and bilateral ANG than HCs. The boxplot showed the distribution of rs-FC values within and between-group (Figure 2B). The edges between ANG and SFGdor were significantly higher than HCs, and an edge between SFGdor and INS was significantly lower in MwoA conditions (FDR-corrected *p*-value lower than 0.05) (Figure 2C).

### Correlation Matrix Calculation and Modules Construction

The correlation matrix showed no significant correlation between significantly altered FC edge and headache duration, frequency, SAS, and SDS. Only one significantly altered edge (rs-FC between dlPFC.L and ANG.L) in the MwoA condition was significantly correlated with headache intensity (Figure 2D). Six edges significantly correlated with headache intensity (SFGdor.L – ANG.L, SFGdor.R – INS.R, MFG.L – MFG.R, MFG.R – ACG.L, ACG.L – IPL.R, and SMG.L – ANG.L), four edges significantly correlated with headache frequency, five edges significantly correlated with headache duration, 12 edges significantly correlated with SAS, and six edges significantly correlated with SDS (Figure 2E). The rs-FC values in edges with ACC (ACG.L – IPL.R and MFG.R – ACG.L) were selected to construct the moderating modules. Before the regression modules estimation, no collinearity was found among the independent variables.

**FIGURE 3:**
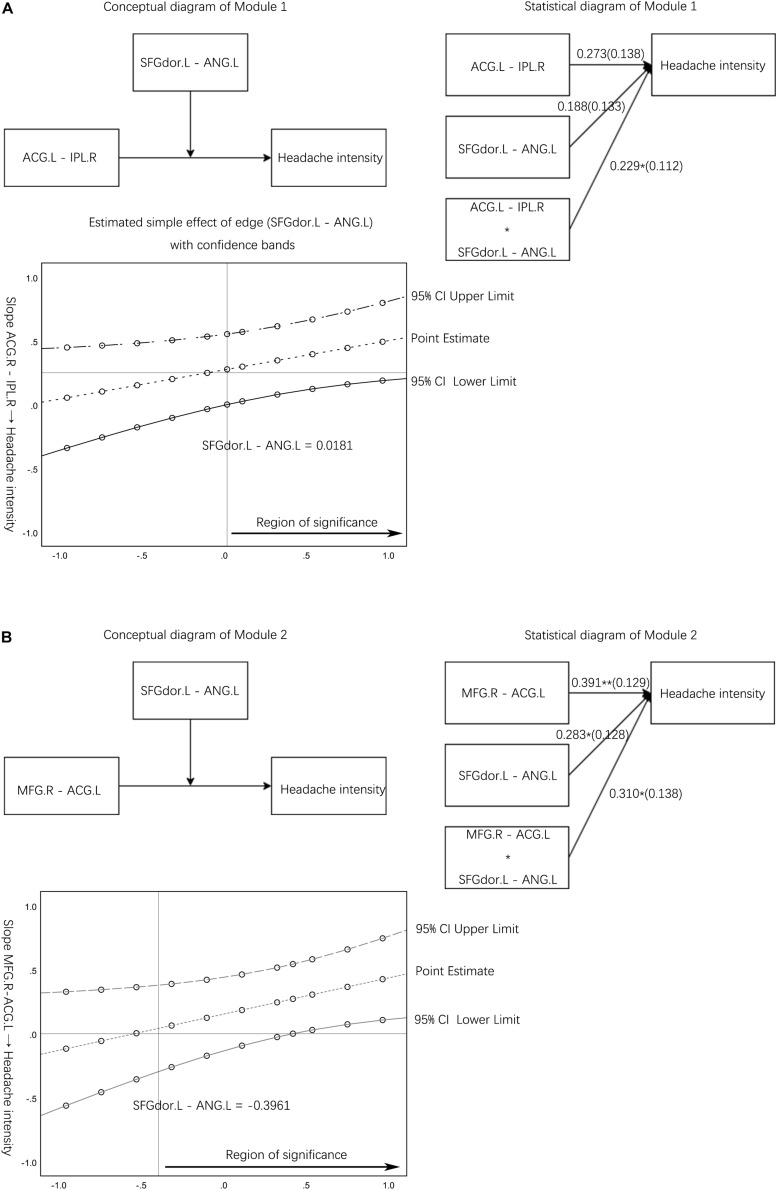
Moderation effect module diagram and simple slope test results. **(A)** The conceptual diagram and statistical diagram of the moderator variable (resting-state functional connectivity edge between SFGdor.L and ANG.L) moderated the link between the edge (ACG.L – IPL.R) and headache intensity. A Johnson-Neyman output plot of the edge (SFGdor.L – ANG.L) indirect effect on the link between the normal edge (ACG.L – IPL.R) and headache intensity. **(B)** The conceptual diagram and statistical diagram of the interaction moderated the link between the edge (MFG.R – ACG.L) with headache intensity. A Johnson-Neyman output plot of the edge (SFGdor.L – ANG.L) indirect effect on the link between the normal edge (MFG.R – ACG.L) and headache intensity. The horizontal line denotes an indirect effect of zero. The vertical line represents the boundary of the region of significance. rs-FC, resting state-functional connectivity; L, left hemisphere; R, right hemisphere; SFGdor, dorsolateral superior frontal gyrus; ANG, angular gyrus; MFG, middle frontal gyrus; ACG, anterior cingulate and paracingulate gyri; IPL, inferior parietal gyrus.

Module 1: the FC edge (SFGdor.L – ANG.L) moderates the link between another edge (ACG.L – IPL.R) in the pain spatial discrimination module and headache intensity (Figure 3A). Module 1 corresponds to hypothesis 1, and the validation of Module 1 can illustrate that clinical symptoms will be affected by one pain discrimination module. Module 2 corresponds to hypothesis 2, which suggests that clinical symptoms will be affected by both spatial and intensity discrimination modules. The FC edge (SFGdor.L – ANG.L) moderates the link between the edge (MFG.R – ACG.L) and headache intensity would be tested to investigate hypothesis 2 (Figure 3B).

### Moderating Effect and Simple Slope Tests

Module 1 manifested the interaction significantly moderated the link between the edge (ACG.L – IPL.R) and headache intensity (*b* = 0.229, *p* < 0.05) (Table 2). The simple slope test demonstrated that the higher level of the altered edge (SFGdor.L – ANG.L) was associated with higher headache intensity (*b*_simple_ = 0.501, *p* < 0.05). However, the low level of the altered edge (SFGdor.L – ANG.L) was not significantly associated with headache intensity (*b*_simple_ = 0.044, *p* > 0.05). The simple slope test provided a perspective on the influence of altered rs-FC edge (SFGdor.L – ANG.L) on pain intensity, indicated that when the MwoA patients have a higher level of altered FC, the related rs-FC edge (ACG.L – IPL.R) affects the headache intensity at a higher level compared with the lower level of altered rs-FC (Figure 3A).

**TABLE 2:** Moderating effect tests of edges between altered resting-state functional connectivity and normal edges on headache intensity.

Predictors	Module 1		Module 2	
	*b*^#^	*t*	*b*^#^	*t*
ACG.L – IPL.R	0.273	1.978		
SFGdor.L – ANG.L	0.188	1.413		
(ACG.L – IPL.R) × (SFGdor.L – ANG.L)	0.229	2.036*		
MFG.R – ACG.L			0.391	3.023**
SFGdor.L – ANG.L			0.283	2.209*
(MFG.R – ACG.L) × (SFGdor.L – ANG.L)			0.310	2.240*
*R*^2^	0.266		0.286	
*F*	5.323**		5.882**	

SFGdor.L, left dorsolateral superior frontal gyrus; ANG.L, left angular gyrus; MFG.R, right middle frontal gyrus; ACG.L, left anterior cingulate and paracingulate gyri; IPL.R, right inferior parietal gyrus; ACG.R, right anterior cingulate; ^#^Standardized Coefficients Beta; *Correlation is significant at the 0.05 level (2-tailed, uncorrected); **Correlation is significant at the 0.01 level (2-tailed, uncorrected).

The *X* and *W* variables were significantly associated with headache intensity, and the *X* variable (edge between MFG.R – ACG.L) is the main effect in Module 2. The significant interaction (interaction of the *X* and *W* variables) manifested the moderator variable *W* (edge between SFGdor.L and ANG.L) moderated the link between the edge (MFG.R – ACG.L) and headache intensity (*b* = 0.310, *p* < 0.05) (Table 2). The simple slope test demonstrated that the higher level of the altered edge (SFGdor.L – ANG.L) was associated with higher headache intensity (*b*_simple_ = 0.701, *p* < 0.05). The low level of the edge (SFGdor.L – ANG.L) was not significantly associated with headache intensity (*b*_simple_ = 0.081, *p* > 0.05). Unlike Module 1, Module 2 was constructed based on moderating effect hypothesis 2, and the simple slope test showed that spatial or intensity discrimination modules have a higher impact when combined with another module on clinical symptoms compared to the single module.

## Discussion

This study firstly investigated the rs-FC pattern of the defined ROIs based on the pain integration hypothesis between MwoA patients and HCs. The ROI-to-ROI analysis demonstrated that MwoA patients had a significantly lower rs-FC between dlPFC.L and INS.L and significantly higher rs-FCs between dlPFC.L and bilateral ANG compared with HCs. The following moderating effect test verified the hypotheses: the pain features integration processes in migraineurs vary from HCs, related to the clinical symptoms during a migraine attack. Moreover, the clinical symptoms will be affected by one or more discrimination modules. And the spatial or intensity discrimination modules have a higher impact when combined with another module on clinical symptoms than the single module.

As the center in both intensity and spatial discrimination modules, ACC receives and integrates input from the dorsal and ventral streams. It is interconnected with prefrontal and parietal, integrates diverse, behaviorally relevant information across multiple timescales (Monosov et al., 2020). When processing input from dlPFC and PPC, ACC is affected by these inputs (Ma et al., 2016). Under these complex mechanisms, it is difficult to investigate the correlation with pain directly. The moderating effect test is an effective approach to explain some of the mechanisms involved.

With the discrimination and integration of the pain sensory features module, both dorsal and ventral streams were integrated to complete the sensation of pain (Figure 1). The bilateral PFC and insula engage in pain intensity discrimination. The PFC can be divided into the mPFC, the orbitofrontal cortex, the ventrolateral PFC (vlPFC), and dlPFC. The loss of PFC gray matter and decreased metabolism would alter the pain modulation processing, manifesting as lower pain thresholds and pain intensity (Niddam et al., 2017). In migraine studies, PFC, especially mPFC and dlPFC, is involved in migraine attacks as a component of DMN and CEN (Seminowicz and Moayedi, 2017; Argaman et al., 2020; Ke et al., 2020). Coppola et al. (2019) suggested that the inability of patients with chronic headaches to allocate relevant attention resources to pain prevents physiological participation in the inactivation mechanism caused by the transition from controlled treatment to the automatic treatment of pain, usually accompanied by DMN inactivation. Also, studies pointed out that dlPFC and mPFC were involved in the pain encoding process (Lev et al., 2013; Hubbard et al., 2014; Schulz et al., 2015; Seminowicz and Moayedi, 2017). These studies supported our finding that migraineurs had a functional abnormality in pain evaluation, which lead to pain sensation abnormalities. The moderating effect tests approved that pain information integration was affected by multiple regions and modules.

Mazzola et al. (2012) reported that various stimuli activated the mid and posterior insula separately, indicating that the insula contributes to sensory discrimination functions. The ventrally directed processing pathway, including the anterior insula, was critical in intensity versus spatial discrimination. The posterior insula is involved in somatosensory and pain perception, and anterior insula activity represents a combination of pain prediction and prediction error (Geuter et al., 2017). The ROI-to-ROI analysis demonstrated that MwoA patients had a significantly lower rs-FC between dlPFC.L and INS.L, representing abnormal pain intensity evaluating module. It suggested that pain intensity discrimination dysfunctions related to the abnormal pain prediction, which insula played a critical role in the appropriate use of cognitive information (Starr et al., 2009; Ploner et al., 2010). The dysfunctions associated with atypical multisensory processing and impaired pain evaluation resulted in sensory hypersensitivity and increased pain intensity during migraine (Ke et al., 2020).

Meanwhile, spatial information could be transferred via a dorsally directed pathway from S1 and the secondary somatosensory cortex (S2) to the PPC and then to the dlPFC (Oshiro et al., 2009). The PPC is divided into the superior parietal lobule (Brodmann areas 5 and 7) and the inferior parietal lobule (Brodmann areas 39 and 40), which participated in selective attention, evidence accumulation, decision making, and working memory (Whitlock, 2017). Although abnormalities in the PPC are quite common in migraine patients (Dumkrieger et al., 2019; Hu et al., 2019; Ke et al., 2020), which critically contributes to sensorimotor integration and is involved in multiple dimensions of information processing ranging, it is difficult to distinguish the specific role of PPC (Freedman and Ibos, 2018; Mohan et al., 2018).

The significantly higher rs-FC increased between dlPFC and bilateral ANG in patients indicated that the MwoA patients had abnormal functions in spatial information discrimination compared with HCs. Koenigs et al. (2009) suggested that SPL is associated with deficits in manipulating and rearranging information within working memory for visual-spatial stimuli. In Modules 1, the moderating effect tests showed a not significant main effect but a significant interaction, suggested that the intensity of pain would be amplificated while integrating the inconsistent pain sensation (Baliki and Apkarian, 2015; Vachon-Presseau et al., 2016).

Moderating effect analysis is used as a critical test for the interaction hypothesis. Thus two moderating effect hypotheses were developed and tested in this study. Unlike the mediating effect analysis, the moderating effect analysis uses hierarchical regression methods that focus on the relationship between the independent variables and the dependent variable. Both Module 1 and 2 in this study showed an increase of the main effect in correlation after hierarchical regressions, which further illustrates the role of altered FC in clinical migraine symptoms. Specifically, clinical symptoms will be influenced by one or more discrimination modules, and multiple modules will be more effective in explaining pain than a single module. Due to the pain being a complex experience, the moderating effect analysis fills the gap of correlation analysis when facing complex modules. In Module 1, where the *X* and *W* variables are not correlated, the interaction terms are significantly correlated. By performing a simple slope test on the interaction with a significant moderation effect, which reflects the moderator’s effect on the main effect, Module 2 has a larger significant interval than Module 1, suggesting that Module 2 has a better predictive effect. The trend of predicted values with increasing rs-FC values had a better estimation in the 95% upper and 95% lower limits, indirectly pointing out that elevated regional synchrony is associated with clinical symptoms of pain.

## Conclusion

In conclusion, this study demonstrated that the dysfunction of discrimination and integration of pain information in MwoA patients affected headache intensity. Furthermore, the information integration function in the spatial or intensity information evaluating module would be affected by another module. The altered function within the spatial or intensity information evaluating module would affect the normal pain information discrimination and integration function. Thus, pain information integration was affected by multiple regions and modules.

## Data Availability Statement

The raw data supporting the conclusions of this article will be made available by the authors, without undue reservation.

## Ethics Statement

The studies involving human participants were reviewed and approved by the Ethics Committee of the 1st Teaching Hospital of Chengdu University of Traditional Chinese Medicine. The patients/participants provided their written informed consent to participate in this study.

## Author Contributions

LL, FZ, and FL conceived and designed the study. QX, XD, YY, MW, GH, and JC recruited the subjects. ZT and TY analyzed the data. ZT drafted the manuscript. LL and FZ revised the manuscript. All authors contributed to the article and approved the submitted version.

## Conflict of Interest

The authors declare that the research was conducted in the absence of any commercial or financial relationships that could be construed as a potential conflict of interest.

## Footnotes

1 https://www.fil.ion.ucl.ac.uk/
