# Supplementary material for: The Altered Functional Connectivity With Pain Features Integration and Interaction in Migraine Without Aura
Source: Front Neurosci. 2021 Mar 4;15:646538. doi: 10.3389/fnins.2021.646538 (PMC7969893; doi:10.3389/fnins.2021.646538)
Supplement: Supplementary file 1 [file Table_1.DOCX]

Supplementary Material

| Labels | Regions | Regions | abbr. | volume-based ROI | | | volume-based ROI | | | number of voxels within ROIs |
| --- | --- | --- | --- | --- | --- | --- | --- | --- | --- | --- |
|  |  |  |  | x(voxel) | y(voxel) | z(voxel) | x(mm) | y(mm) | z(mm) |  |
| 3 | Frontal_Sup_L | Superior frontal gyrus, dorsolateral | SFGdor.L | 71.55431 | 160.8081 | 114.2017 | -18.4457 | 34.8081 | 42.2017 | 1076 |
| 4 | Frontal_Sup_R | Superior frontal gyrus, dorsolateral | SFGdor.R | 111.8997 | 157.1201 | 115.8154 | 21.8997 | 31.1201 | 43.8154 | 1159 |
| 7 | Frontal_Mid_L | Middle frontal gyrus | MFG.L | 56.5682 | 158.7318 | 107.4556 | -33.4318 | 32.7318 | 35.4556 | 1448 |
| 8 | Frontal_Mid_R | Middle frontal gyrus | MFG.R | 127.5948 | 159.0619 | 106.0351 | 37.5948 | 33.0619 | 34.0351 | 1510 |
| 29 | Insula_L | Insula | INS.L | 54.86902 | 132.6518 | 75.44093 | -35.131 | 6.6518 | 3.4409 | 566 |
| 30 | Insula_R | Insula | INS.R | 129.0191 | 132.2451 | 74.07751 | 39.0191 | 6.2451 | 2.0775 | 539 |
| 31 | Cingulum_Ant_L | Anterior cingulate and paracingulate gyri | ACG.L | 85.96342 | 161.3999 | 85.95287 | -4.0366 | 35.3999 | 13.9529 | 426 |
| 32 | Cingulum_Ant_R | Anterior cingulate and paracingulate gyri | ACG.R | 98.45566 | 163.0056 | 87.83978 | 8.4557 | 37.0056 | 15.8398 | 397 |
| 59 | Parietal_Sup_L | Superior parietal gyrus | SPG.L | 66.5464 | 66.44276 | 130.9601 | -23.4536 | -59.5572 | 58.9601 | 631 |
| 60 | Parietal_Sup_R | Superior parietal gyrus | SPG.R | 116.1058 | 66.82015 | 134.0618 | 26.1058 | -59.1798 | 62.0618 | 647 |
| 61 | Parietal_Inf_L | Inferior parietal, but supramarginal and angular gyri | IPL.L | 47.19628 | 80.18034 | 118.7405 | -42.8037 | -45.8197 | 46.7405 | 696 |
| 62 | Parietal_Inf_R | Inferior parietal, but supramarginal and angular gyri | IPL.R | 136.462 | 79.7103 | 121.5351 | 46.462 | -46.2897 | 49.5351 | 419 |
| 63 | SupraMarginal_L | Supramarginal gyrus | SMG.L | 34.21187 | 92.36217 | 102.4495 | -55.7881 | -33.6378 | 30.4495 | 354 |
| 64 | SupraMarginal_R | Supramarginal gyrus | SMG.R | 147.6107 | 94.49512 | 106.48 | 57.6107 | -31.5049 | 34.48 | 550 |
| 65 | Angular_L | Angular gyrus | ANG.L | 45.85869 | 65.18415 | 107.5872 | -44.1413 | -60.8158 | 35.5872 | 342 |
| 66 | Angular_R | Angular gyrus | ANG.R | 135.5077 | 66.01535 | 110.6277 | 45.5077 | -59.9847 | 38.6277 | 496 |

**Supplementary 1.** The table of defined ROIs addressed by automated anatomical labeling atlas included bilateral dorsolateral superior frontal gyrus, bilateral middle frontal gyrus, bilateral insula, bilateral anterior cingulate and paracingulate gyri, bilateral superior parietal gyrus, bilateral inferior parietal gyrus, bilateral supramarginal gyrus, bilateral angular gyrus.


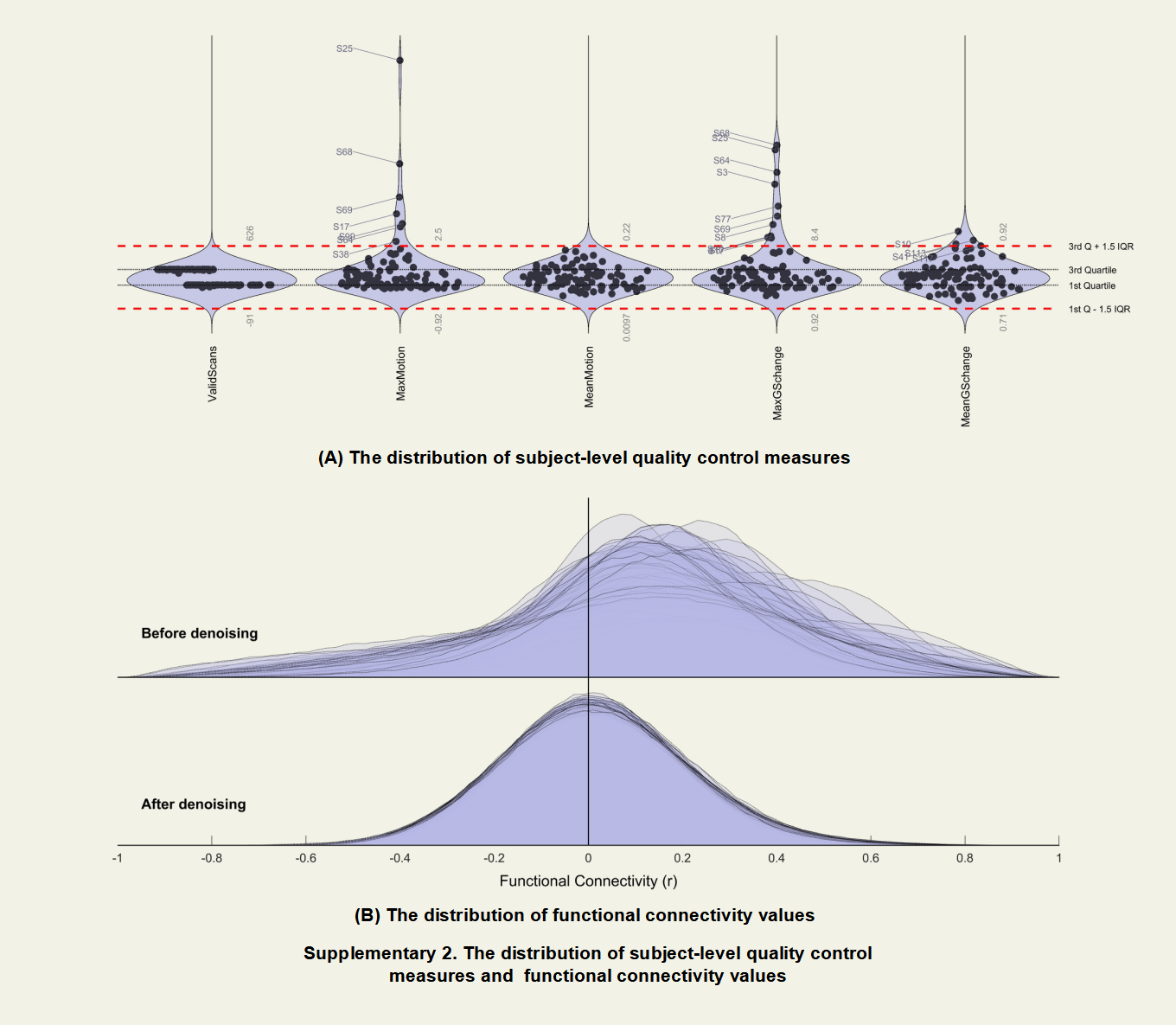


**Supplementary 2.** Quality assurance was checked at the subject-level. (A) showed that all scans were valid. (B) showed the denoising process improved comparability at the subject-level.
